# Increasing The Genetic Admixture of Available Lines of Human Pluripotent Stem Cells

**DOI:** 10.1038/srep34699

**Published:** 2016-10-06

**Authors:** Fabiano A. Tofoli, Maximiliano Dasso, Mariana Morato-Marques, Kelly Nunes, Lucas Assis Pereira, Giselle Siqueira da Silva, Simone A. S. Fonseca, Roberta Montero Costas, Hadassa Campos Santos, Alexandre da Costa Pereira, Paulo A. Lotufo, Isabela M. Bensenor, Diogo Meyer, Lygia Veiga Pereira

**Affiliations:** 1 National Laboratory for Embryonic Stem Cells (LaNCE), Department of Genetics and Evolutionary Biology, Biosciences Institute, University of São Paulo, SP 05508-900, Brazil; 2 Laboratory of Evolutionary Genetics, Department of Genetics and Evolutionary Biology, Biosciences Institute, University of São Paulo, SP 05508-900, Brazil; 3 Instituto do Coração, Hospital das Clínicas da Faculdade de Medicina, Universidade de São Paulo. Av. Dr. Enéas de Carvalho Aguiar, 44, São Paulo, SP 05403-900, Brazil; 4 Center of Clinical and Epidemiologic Research, University Hospital, University of São Paulo, SP 05508-000. Brazil

## Abstract

Human pluripotent stem cells (hPSCs) may significantly improve drug development pipeline, serving as an *in vitro* system for the identification of novel leads, and for testing drug toxicity. Furthermore, these cells may be used to address the issue of differential drug response, a phenomenon greatly influenced by genetic factors. This application depends on the availability of hPSC lines from populations with diverse ancestries. So far, it has been reported that most lines of hPSCs derived worldwide are of European or East Asian ancestries. We have established 23 lines of hPSCs from Brazilian individuals, and we report the analysis of their genomic ancestry. We show that embryo-derived PSCs are mostly of European descent, while induced PSCs derived from participants of a national-wide Brazilian cohort study present high levels of admixed European, African and Native American genomic ancestry. Additionally, we use high density SNP data and estimate local ancestries, particularly those of CYP genes loci. Such information will be of key importance when interpreting variation among cell lines with respect to cellular phenotypes of interest. The availability of genetically admixed lines of hPSCs will be of relevance when setting up future *in vitro* studies of drug response.

Human pluripotent stem cells (hPSCs) are an ideal cell source for the development of cell based assays for drug response. In addition to their extensive proliferation and genetic stability in culture, these human cells can give rise to primary cell types relevant for drug response, including cardiomyocytes, hepatocytes and neurons^1^. Individual differences in drug response can result from the effects of age, sex, disease, ancestry, or drug interactions, but genetic factors play a major role in influencing adverse drug reactions and ineffective therapy^2^. Thus, a collection of genetically diverse lines of hPSCs is required for a broader study of differential drug response *in vitro*^3^.

To date, most lines of hPSCs available are of European or Eastern Asian ancestry^4^, although one hiPSC line from a Native American, and one from an African (Yoruba) have been reported^5^. More recently, one hiPSC line of African American and of Hispanic Latino ancestry each have been described, although the authors do not show the genetic evidence of those ethnicities^6^.

The Brazilian population results from 500 years of admixture among the original Native Americans, Europeans (mostly Portuguese), and sub-Saharan Africans, most of which were brought to the country as slaves^7^. Different analyses of genomic ancestry in Brazil have shown that, on average, the urban population has 60% contribution from European, 25% from African, and 15% from Native American populations, although these proportions vary according to the Brazilian geographic region analyzed^7^,^8^,^9^,^10^,^11^. Therefore, from the genetic point of view the Brazilian population is significantly distinct from the ancestral populations, containing novel genotypes and haplotypes that may impact various phenotypes, including drug response.

We have previously established five lines of hPSCs from surplus human embryos generated for reproduction purpose and stored in private human reproduction clinics in Brazil^12^,^13^,^14^. Here we report the derivation of 18 lines of hPSCs from peripheral blood of participants of The Brazilian Longitudinal Study of Adult Health (ELSA-Brasil), a large multicenter cohort study of 15,105 Brazilians, focused on assessing incidence and risk factors for diabetes and cardiovascular diseases in the country^15^,^16^. We analyze the genomic ancestry of the different hPSC lines, and show that while the lines derived from human embryos (human embryonic stem cells, hESCs) are mostly of European genomic descent, those derived from the participants of the ELSA-Brasil present a broader range of ancestries, providing a better reflection of the genetic admixture that characterizes the Brazilian population. The hiPSCs described here significantly increase the spectrum of genetic admixture of the available lines, and can be an important resource for the study of the molecular basis of differential drug response.

## Results and Discussion

### Genomic ancestry of hPSCs

Mononuclear cells from peripheral blood of 1,872 participants of the ELSA study were isolated and cryopreserved. Eighteen of those were randomly selected for hiPSC derivation. Reprogrammed colonies were pooled and expanded for pluripotency evaluation. Cell lines were shown to have normal karyotype (data not shown), homogeneously express the pluripotency markers OCT4, NANOG and SSEA-4, and to differentiate *in vitro* into tissues from the three embryonic germ layers (Supplemental Fig. 1). In addition, PCR analysis showed no integration of the reprogramming vectors, and thus the hiPSC lines are footprint-free (Supplemental Fig. 1).

**Figure 1:**
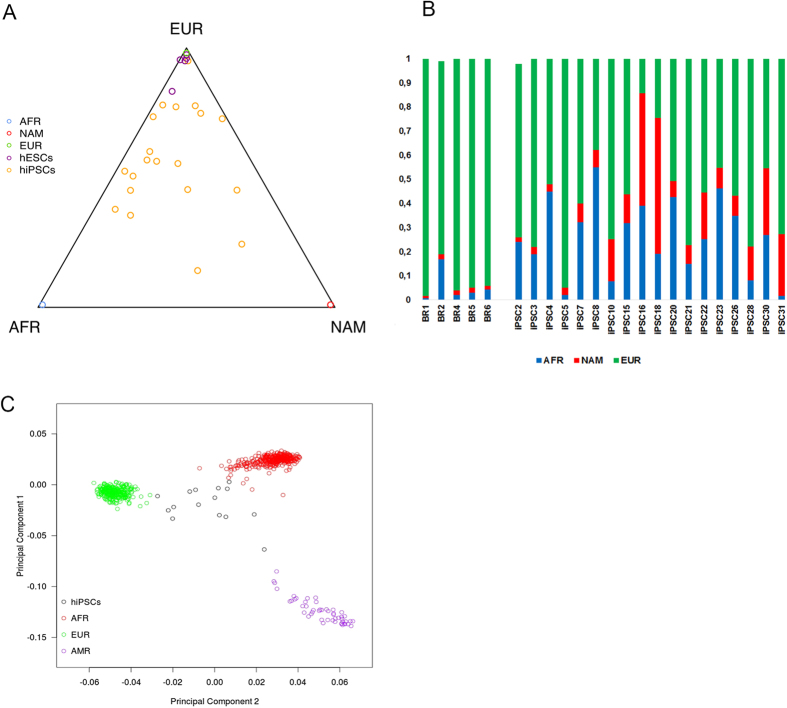
Genomic ancestry of hPSCs. (**A**) Ternary plot of African, European, and Native American ancestry in hPSC lines. Each yellow point represents hiPSC lines, purple points represents hESC lines. Each point is positioned within the triangle reflecting the amount of ancestry estimated from each cell line; (**B**) Percentage of the contributions of different ancestral genomes of each hPSCS; (**C**) Principal component (PC) analysis of the hiPSC lines. PC1 and PC2 are plotted in the x and y axes, respectively. Comparison populations from the HGDP and HapMap are shown in green (European), red (Africans) and purple (Native American). hiPSCs are represented by black circles.

Genomic ancestries of five lines of hESCs derived from embryos generated in Brazil for reproductive purpose and of the eighteen hiPSCs were determined by the analysis of 192 ancestry informative markers and comparison with reference populations (Fig. 1). The data showed that Brazilian hESC lines are mostly of European ancestry, with the European genomic component ranging from 92.7% to 98.6%. In contrast, in the 18 randomly chosen lines of hiPSCs from the ELSA-Brasil, the European genomic contribution ranged from 14.2% to 95%, while African ancestry ranged from 1.6% to 55.1%, and Native American ancestry ranged from 7% to 56% (Fig. 1a,b). Principal component analysis (PCA) of hiPSCs showed that most of them are on the African - European variation axis (Fig. 1c).

### Local ancestry of hPSCs around CYP genes

**Figure 2:**
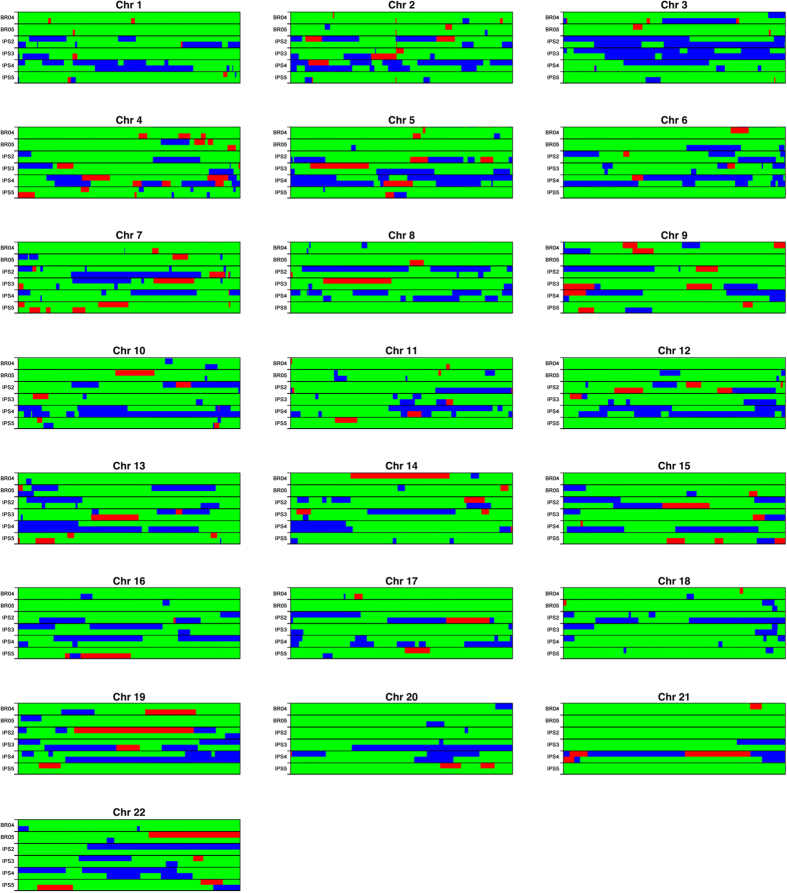
Local ancestry estimative in two hESC (BR-4 and BR-5) and four hiPSC lines (IPS2, IPS3, IPS4, IPS5). Each row is a chromosome sample and every two consecutive rows is a pair of individual chromosomes. The column represents the SNPs and the colors represent the ancestry: Blue – African, Green –European, Red – Native American.

**Figure 3:**
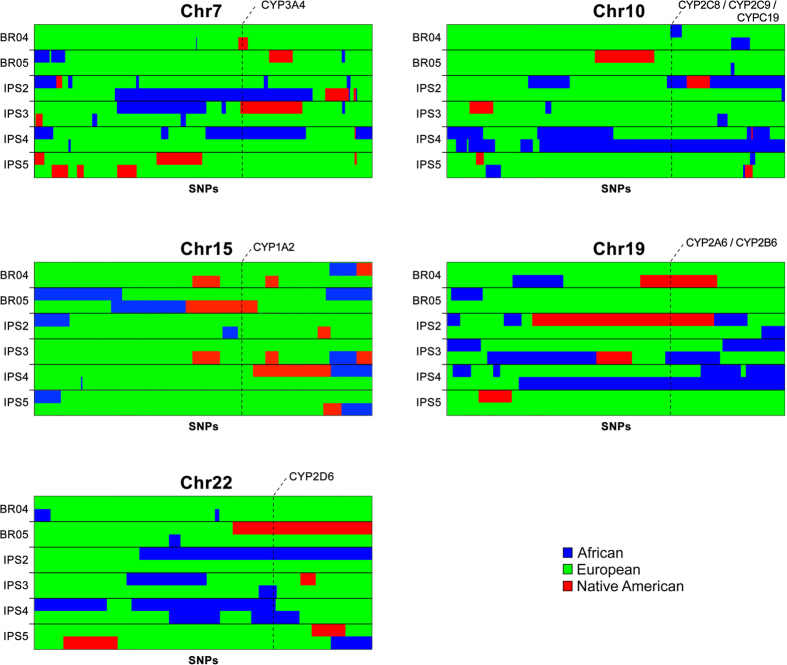
Local ancestry of hPSCs around CYP genes. Local ancestry estimative for CYP family genes in two hESC (BR-4 and BR-5) and four hiPSC lines (IPS2, IPS3, IPS4, IPS5). Each row is a chromosome sample and every two consecutive rows is a pair of individual chromosomes. The column represents the SNPs and the colors represent the ancestry: Blue – African, Green – European, Red – Native American. The horizontal dashed lines indicate the location of the CYP gene indicated.

We randomly chose two hESCs and four hiPSCs to perform a local ancestry analysis, i.e., to determine the ancestry for each physical location in the genome. As expected under a scenario of admixture that started several generations ago, the chromosomes of the hPSC lines analyzed (hESCS: BR-4 and BR-5, and hiPSCS IPS2, IPS3, IPS4 and IPS5) are mosaics comprised of segments of the three ancestries (Fig. 2). We investigated the local ancestry of specific genes belonging to the Cytochrome P450 (CYP) family (*CYP1A2, CYP2C8, CYP3A4, CYP2C9, CYP2C19, CYP2D6, CYP2B6, CYP2A6*), known for its role in drug metabolism. We found a high level of heterogeneity of ancestry among the hPSC lines, with all CYP genes investigated having cell lines with representatives from African and European ancestries, and all loci except *CYP2C8*/*CYP2C9*/*CYP2C19* having at least one instance in which it lies in a region of Native American ancestry (Fig. 3).

Human PSCs are a valuable model system in which to study Mendelian conditions, where a single gene mutation triggers the clinical phenotype^17^. The use of these cells to model complex disorders, where the phenotype results from the interaction of multiple mutations and environmental factors, is far more challenging^18^. In particular, differential drug response has been shown to be greatly influenced by genetic factors. Thus, collections of hPSCs with different genetic backgrounds must be used to dissect the molecular basis and to develop cell based assays of differential drug toxicity and efficacy. Nevertheless, three studies of genomic ancestry have shown limited ethnic diversity of the available lines of hPSCs, the great majority being of European and Asian ancestry^4^,^5^,^19^. Importantly, none of those lines derived from populations with recent African ancestry^19^.

Analysis of the genomic ancestry of all lines of hESCs derived in Brazil confirmed our initial hypothesis that the embryos available for research do not represent the ethnic diversity of the Brazilian population^12^. While the European contribution to the Brazilian population ranges from 37% to 82%^20^, the five Brazilian lines of hESCs were 92% to 98% of European genomic ancestry. In contrast, eighteen hiPSCs analyzed here presented a broad range of genomic ancestries, with the European contribution ranging from 14.2% to 95.0%. In addition, the PCA shows that the ancestry variability of the hiPSCs is mainly between the European and African components. Thus, we were able to find high genetic admixture in hiPSCs derived from participants of the ELSA-Brasil cohort study.

Moreover, we also analyzed the ancestry of 2 hESC and 4 hiPSC in the region containing eight genes from the CYP family, responsible for the metabolism of the majority of drugs and xenobiotics. We looked specifically at CYP family members that have polymorphisms described in different frequency between the ethnic groups, and different levels of metabolic response reported^21^. We showed the analyzed hPSC have great heterogeneity of ancestry in the regions of CYP genes. We also showed that although there is a predominance of European ancestry genomewide, different ancestries are often present in the genomic region of interest, including the occurrence of Native American ancestry for CYP genes in otherwise predominantly European genomes. This genetic heterogeneity is of great interest, since possible genetic variants of specific ancestry may be present and influence the functionality of gene products related to differential drug response and other complex phenotypes.

The ELSA-Brasil cohort study represents a unique opportunity for the generation of collections of hiPSCs for different research purposes. The study collects data from 15,000 35–74 year old participants from three regions of Brazil (Northeast, Southeast and South) every 3–4 years in the form of interviews, electrocardiogram, blood sample, blood pressure, as well as other clinical, biochemical and genetic tests^15^. Base-line findings identified several interesting clinical groups, including diabetes (19.7%), common mental disorders (26.7%) and coronary heart disease (4.7%)^16^. Thus, while a collection of genetically admixed hiPSCs can be generated from these participants for future *in vitro* clinical trials and other studies of drug response, the availability of extensive clinical data of each participant permits the generation of different collections of cells based on phenotypes of interest. For example, a group of 4,116 participants being treated for hypertension was identified, 11% of those being resistant to pharmacological intervention^22^ – we are currently generating a collection of hiPSCs from responsive and resistant hypertensive individuals to understand and to predict resistant hypertension.

## Conclusions

We derived lines of hiPSCs with novel combinations of genomic ancestry, including Native American and African. In addition, we have established a collection of primary cells from a well clinically characterized population ready to be reprogrammed into hiPSCs for different research purposes. The new hiPSC lines described here significantly increase the ethnic diversity and genetic admixture of the currently available hPSCs.

## Methods

### Subjects

Participants of the ELSA-Brasil in São Paulo were invited to participate in this research. Exclusion criteria were current or recent (<4 months prior to the first interview) pregnancy, intention to quit working at the institution in the near future, severe cognitive or communication impairment, and, if retired, residence outside of a study center’s corresponding metropolitan area. The first examination was carried out from 2008 through 2010. Annual telephone surveillance for outcomes is now in its seventh year, and the first follow-up examination was conducted form 2012 through 2014. From the 5061 participants, 1872 were randomly chosen to be included in the present analysis. Participants signed informed consents. The project was carried out in accordance with the guidelines for research on human subjects, and approved by the Ethics Committee of the University Hospital, University of São Paulo.

### Human PSC lines

Human embryonic stem cell lines BR-1, BR-2, BR-4, BR-5 and BR-6 were cultured as described^12^,^13^,^14^. Human iPSC lines were derived from erythroblasts with episomal vectors using a protocol described^23^,^24^ with modifications. Briefly, mononuclear cells were isolated from 10 ml of peripheral blood by Ficoll gradient. Erythroblasts were cultured in a serum-free mononuclear cell (MNC) medium containing the following cytokines diluted in Stem Span Serum Free Expansion Medium (Stem Cell Technologies, Cat No. 09650): insulin-like growth factor 1 (IGF-1): 40 ng/ml; Stem Cell Factor (SCF): 100 ng/ml; Interleukin 3 (IL-3):10 ng/ml; erythropoietin (EPO): 2 U/ml. Two million cells were transfected with plasmids pEB-C5 and pEB-Tg (Addgene), containing reprogramming factors *Oct4, Sox2, Klf4, cMyc, Lin28* and *SV40-T*, using the Human CD34+ nucleofector kit and the Nucleofector II device, both by Lonza (Basel, Switzerland) following manufacturer’s instructions. Reprogrammed erythroblasts were incubated in MEF-coated plates in MEF medium and FBS ES-Cell Qualified (ESQ, Invitrogen) with basic fibroblast growth factor (bFGF; 20 ng/ml) overnight. Then, they were transferred into embryonic stem cell (ESC) medium containing Knockout DMEM (Life Technologies), Knockout Serum replacement (Life Technologies, Catalog No. 10828–028), Antibiotic-antimycotic (Life Technologies), Glutamax 200 mM (Life Technologies), MEM non‐essential amino acid solution (Life Technologies), 2‐mercaptoethanol (Life Technologies) supplemented with bFGF (20 ng/ml) and Sodium butyrate (0.25 mM). hiPSC colonies were passaged from a 6 well MEF-coated plate into Matrigel (BD)-coated plates with E8 medium (Invitrogen), using Gentle Cell Dissociation Reagent (Stemcell Technologies) and 10 μM ROCK inhibitor Y-27632 (Stemgent).

### Flow Cytometry

Cells were fixed and labeled using *Human Pluripotent Stem Cell Transcription Factor Analysis Kit* from BD according to the manufacturer’s instructions. Cells were incubated with human monoclonal antibodies specific to Nanog-PE, Oct3/4-PerCP-Cy5.5 and Alexa 647-Sox2 and corresponding isotype controls for 30 minutes and washed twice with PBS. Fifty thousand events were acquired in *BD Accuri C6 flow cytometer* using the kit template. Cells were gated on light scatter properties and analyzed for expression of key pluripotency transcription factors using *BD Accuri C6 software*.

### Immunocytochemistry

Cells from passages 6 and 29 were fixed and immunostained following standard protocols^23^. Primary antibodies used were: monoclonal anti-mouse stage-specific embryonic antigen-1 (SSEA-1) and -4 (SSEA-4), TRA-1-60, TRA-1-81 (dilution 1:50; Chemicon), and anti-mouse OCT-4 (dilution 1:100; Santa Cruz Biotechnology, Inc.). Human foreskin fibroblasts (HS27 cell line, ATCC) were used as negative controls. Images were analyzed in an Axiophot 2 fluorescent microscope (Carl Zeiss) and captured by CCD camera using the ISIS software (MetaSystem).

### *In Vitro* Differentiation

To demonstrate spontaneous *in vitro* differentiation, hiPSC and hESC were grown to confluency and harvested by dispase. Cells were washed and re-suspended twice in E8 medium and transferred to non-tissue culture treated 6 well plates coated with 1% agarose. Day 5 embryoid bodies (EBs) were transferred to a 6 well plate coated with 0.1% gelatin, and cultured for a further 15 days in differentiation medium [DMEM, 20% FBS (ESQ), 2 mM L-glutamax, MEM-NEAA 2 mM, 0.1 mM β-mercaptoethanol, 1% HEPES, 1% Sodium piruvate. 1% penicillin-streptomicin (Life Tech)]. Differentiated cells were harvested with Trizol and RNA extraction was performed usingRNeasy kit (Qiagen). One mg of RNA was used for subsequent reverse transcriptase reactions using *High Capacity cDNA* kit (Life Technologies). Pluripotency and trilineage differentiation potential was assessed using the TaqMan^®^ hPSC Scorecard™ kit 384w following manufacturer’s instructions and run on a *StepOne Plus System* (Life Technologies), as described^25^. Data analysis was performed using the cloud based TaqMan^®^ hPSC Scorecard™ analysis software (Life Technologies).

### Vector integration analysis

Absence of vector DNA integration into hiPSC lines was confirmed by PCR analysis as described^16^. Briefly, primers for the Epstein–Barr nuclear antigen 1 (EBNA1) gene region of the pCEP4 vector were used to amplify 150 ng of hiPSC DNA and 3 pg of vector DNA (positive control) for 30 cycles.

### Genomic ancestry

Analysis of genomic ancestry was conducted using the Admixture program^26^. Admixture is a software tool for maximum likelihood estimation of individual ancestries from multilocus SNP genotype datasets. Specifically, Admixture uses a block relaxation approach to alternately update allele frequency and ancestry fraction, with parameter standard errors using bootstrapping. We used a supervised approach for ancestry determination^27^, with 200 bootstrap replicates (default) and k = 3 (number of parental populations assumed for the analysis). The analysis was done using 192 Ancestry Informative Markers developed by our group^28^, genotyped by OpenArray^®^ Real-Time PCR (Applied Biosystems). We used as reference ancestral populations those from the Human Genome Diversity Project (HGDP) (Center SHG. Human Genome Diversity Project): Pima, Maya as Native Americans; and from HapMap project (International HapMap Project): Africans - YRI (Yoruba in Ibadan, Nigeria), LWK (Luhya in Webuye, Kenya), ASW (Americans of African Ancestry in SW, USA); European - CEU (Utah Residents (CEPH) with Northern and Western European ancestry) and TSI (Tuscan in Italia).

Local ancestry analysis was performed by genotyping samples with the Axiom Human Origins Array (approximately 600,000 SNPs) according to manufacturer’s instructions (Affymetrix). As parental populations we used a sample of 30 unrelated African and 30 unrelated Europeans from 1000 Genomes Project (1000 g) and the 30 unrelated Native Americans from the Centre d’Étude du Polymorphisme Humain and the Human Genetic Diversity Panel (CEPH-HGDP). The SNPs from these three datasets (1000 g+HGDP and present study) were filtered and paired according to Nunes *et al*.^29^ Local ancestry analyses were performed using the RFMix software^30^ via a discriminative modeling approach based on a Random Forest algorithm. We used an admixture model that assumed 8 generations since the start of admixture and windows of 0.2 cM. The genotypes were phased using the SHAPEIT software^31^.

## Additional Information

**How to cite this article**: Tofoli, F. A. *et al*. Increasing The Genetic Admixture of Available Lines of Human Pluripotent Stem Cells. *Sci. Rep.* **6**, 34699; doi: 10.1038/srep34699 (2016).

## Supplementary Material

Supplementary Information
