# Supplementary material for: Increasing The Genetic Admixture of Available Lines of Human Pluripotent Stem Cells
Source: Sci Rep. 2016 Oct 6;6:34699. doi: 10.1038/srep34699 (PMC5052616; doi:10.1038/srep34699)

INCREASING THE GENETIC ADMIXTURE OF AVAILABLE LINES OF HUMAN PLURIPOTENT STEM CELLS

Fabiano Tofoli de Araujo, Maximiliano Dasso, Mariana Morato-Marques, Kelly Nunes, Lucas Assis Pereira, Giselle Siqueira da Silva, Simone Fonseca, Roberta Montero, Hadassa Campos Santos, Alexandre da Costa Pereira, Paulo A. Lotufo, Isabela M. Bensenor, Diogo Meyer & Lygia Veiga Pereira.

Supplemental Figure 1: Characterization of pluripotency of hiPSCs from the Brazilian population. (A) Flow cytometry of hiPSC line IPSC3 with antibodies against Nanog, Oct4 and Sox2 (red lines). Percentages of positive cells are indicated. Black lines represent signal from secondary antibody alone. (B) Percentage of cells of different lines of hiPSCs positive for each marker by flow cytometry analysis. Representative (C) ScoreCard analysis of hiPSCs; and (D) PCR analysis of vector integration. Genomic DNA from the indicated hiPSCs were amplified with primers for the EBNA-1 region of plasmid pCEP4 as described[16]. CT – negative control; pEB-C5, pEB-Tg and pEB-SK reprogramming vectors[16] ; MW – 50bd DNA Ladder (Thermofisher).

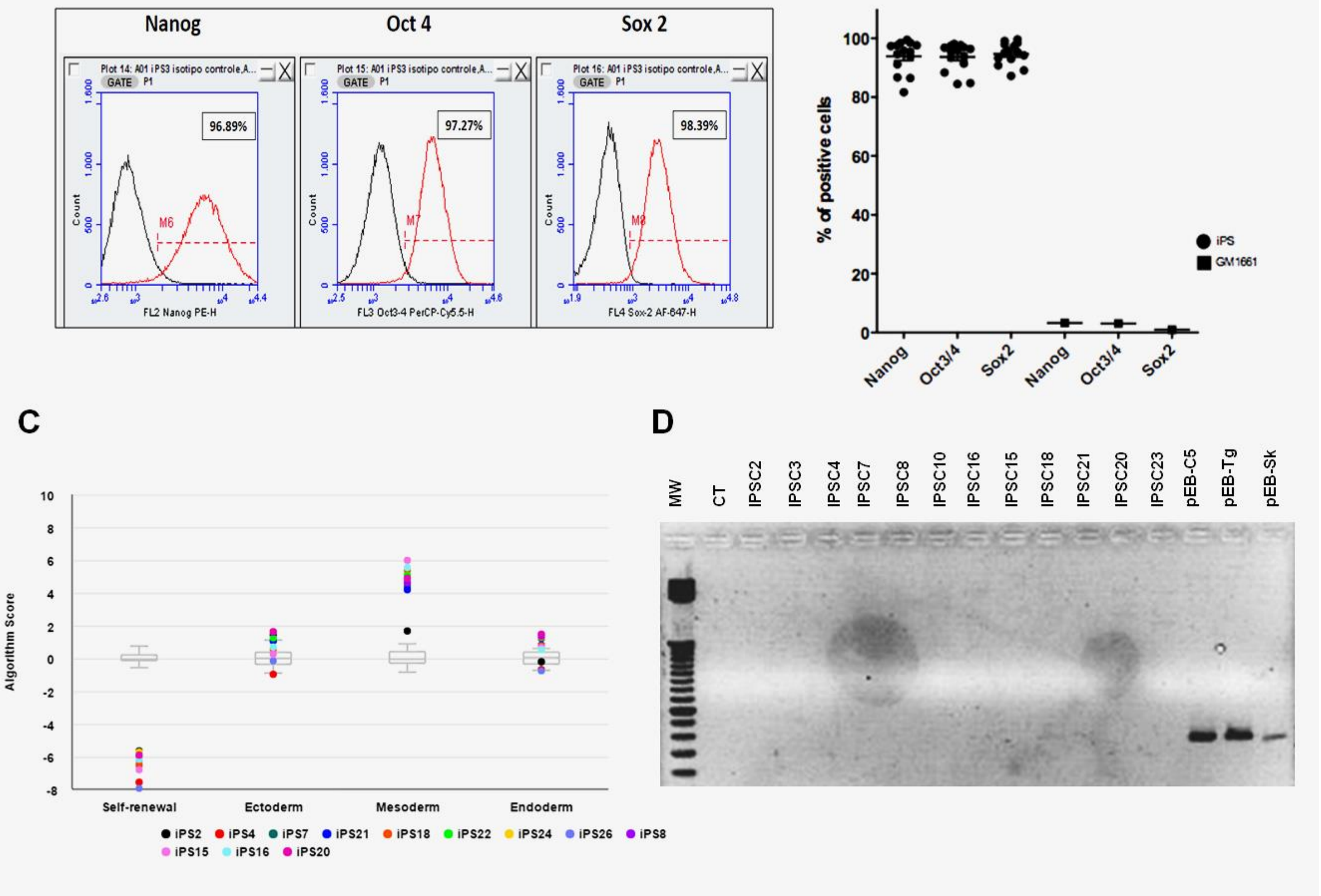

Supplement: Supplementary Information [file srep34699-s1.pdf]
